# Microstructure and Properties of Cu-Fe Immiscible Coatings Fabricated via Combined Mechanical Alloying and Laser Cladding

**DOI:** 10.3390/ma18194436

**Published:** 2025-09-23

**Authors:** Cheng Deng, Tao Xie, Zihao Wan, Guangjian Feng, Yuanlun Yang, Zhaozhi Wu, Xinhua Wang, Shengfeng Zhou, Jie Chen

**Affiliations:** 1 College of Mechatronics Engineering, Guangdong Polytechnic Normal University, Guangzhou 510635, China; dengcheng@gpnu.edu.cn (C.D.); 18279905513@163.com (T.X.); 13022086164@163.com (Z.W.); fengguangjian0629@163.com (G.F.); 15718184987@163.com (Y.Y.); wuzhaozhi@gpnu.edu.cn (Z.W.); wangxh@gpnu.edu.cn (X.W.); 2 Institute of Advanced Wear & Corrosion Resistant and Functional Materials, Jinan University, Guangzhou 510632, China; zhousf1228@163.com; 3 School of Intelligent Manufacturing, Guangzhou Maritime University, Guangzhou 510725, China; 4 South China Robotics Innovation Research Institute, Foshan 510250, China

**Keywords:** laser cladding, mechanical alloying, Cu–Fe immiscible composite coatings, microstructure, comprehensive performances

## Abstract

This work reports on a systematic investigation of the microstructure and comprehensive performance of Cu–Fe immiscible composite coatings prepared through the combination of mechanical alloying and laser cladding. The samples were characterized by scanning electron microscopy with an energy dispersive analysis, X-ray diffraction, a digital microhardness tester, a current tester, an electrochemical analyzer, and a magnetometer. The results show that the immiscible composite coatings are mainly composed of *α*-Fe particle dispersion in the *ε*-Cu matrix due to liquid phase separation, and this is exacerbated by the addition of more Fe content. Concentrated distribution of Fe-rich particles at either the top or bottom of the immiscible composite coatings is driven by the dominant mechanism of Marangoni and Stokes motion. With the increased fraction of Fe content, the microhardness and electrical resistivity increased, but with a degradation in corrosion resistance. With the increased ball milling time, the electrical resistivity increased, and the corrosion resistance improved. Compared to the medium-carbon steel substrate, the immiscible composite coatings can achieve an improved corrosion resistance, as well as a maximum saturated magnetization of 10.172 emu/g and the lowest coercivity at 17.249 Oe.

## 1. Introduction

Applying metal coating materials to substrate surfaces significantly enhances corrosion resistance, wear resistance, and electrical conductivity or imparts oxidation resistance and special appearance characteristics, finding extensive application in fields such as aerospace component protection and automotive part reinforcement. Due to their synergistic multi-phase architecture and exceptional properties, as well as microstructural stability, Cu-based and Fe-based immiscible alloys have attracted significant interest in a rich variety of potential applications, such as electronic packaging solders and magneto-resistive materials in the electronic industry, advanced bearings in the automotive industry, and metallic phase change materials (PCMs) in latent heat storage systems [1,2,3,4,5,6]. However, the mutual solubility between Cu and Fe in the liquid state is very limited; Cu–Fe melt tends to undergo liquid phase separation during solidification: a Cu-rich melt and a Fe-rich melt. This results in severe macro-segregation, inhomogeneous structures, and reduced properties [7,8,9,10]. Thus, controlling liquid phase separation is essential to achieving uniform structures and enhancing the overall performance of Cu–Fe immiscible alloy.

To address the challenge of liquid phase separation between Cu and Fe, extensive work [11,12,13] has been reported on the preparation methods for Cu–Fe immiscible alloy, such as rapid solidification, mechanical alloying, and laser-based methods. Though gravity-driven microstructural segregation can be mitigated by microgravity adjustment, the rapid growth and coalescence of minority-phase droplets are still difficult to suppress due to the persistent influence of Marangoni-driven convection [14,15]. Compared to the above-mentioned methods, laser cladding can be considered an innovative and viable technique to produce Cu–Fe immiscible composite coatings due to many advantages, including rapid heating and cooling rates, refined microstructure, low dilution, controllable composition, minimal thermal distortion, and high bonding strength [16,17,18,19].

Relevant studies [20,21] show that rapid solidification by laser cladding can effectively suppress undesirable liquid phase separation tendencies, enhance the uniformity of phase distribution, and stabilize the resulting microstructure. As a result, controllable composition and enhanced mechanical, thermal, and wear properties can be achieved [22,23,24]. To further optimize the microstructure and interfacial characteristics of precursor powders, mechanical alloying has been introduced as a pre-treatment step before the laser cladding process, which leads to a significant refinement of powders and uniform elemental distribution, facilitating the interfacial bonding and diffusion. For example, He et al. proved that mechanical alloying can significantly improve the laser cladding formability of the refractory high-entropy alloy CoCrMoNbTi [25]. Compared to other powder preparation methods, such as chemical treatment and heat treatment, mechanical alloying enhances the uniformity of powder composition distribution while being simpler and more economical.

In previous studies, it was found that Cu95Fe5 immiscible composite coatings have improved hardness, corrosion resistance, and performance compared to brass [26], but the effect of compositional changes on the performance of Cu-X immiscible composite coatings has not been considered. Therefore, in this work, a systematic experimental study was undertaken to examine the effect of element modification and the processing parameters of mechanical alloying and laser cladding on Cu–Fe immiscible composite coatings. Microstructure modification and hardness improvement were studied by scanning electron microscopy and micro-indentation, respectively. Electrical resistivity, electrochemical corrosion, and magnetic properties were also examined by a current conductivity meter, an electrochemical analyzer, and a magnetometer, respectively.

## 2. Materials and Methods

### 2.1. Materials

**Figure 1:**
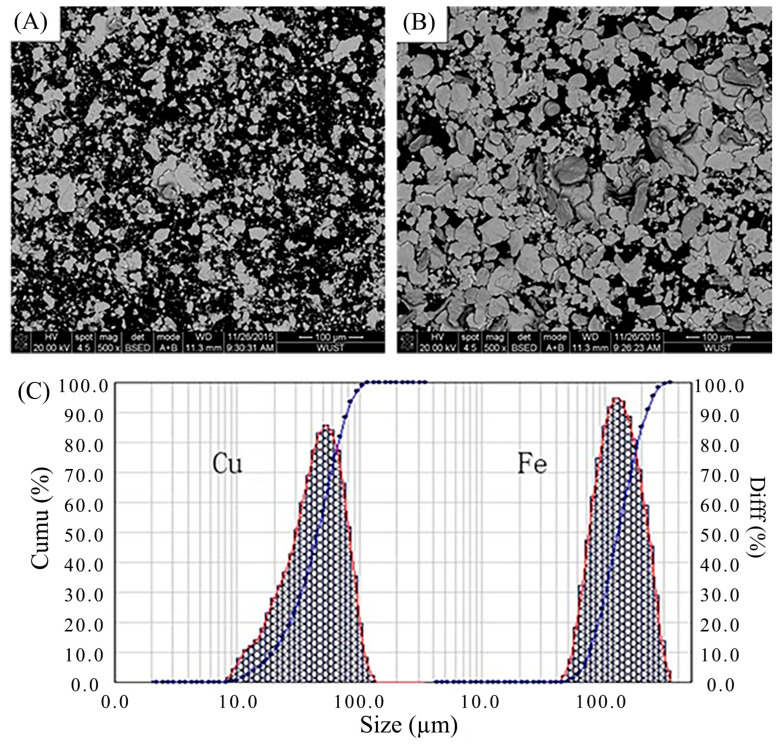
SEM morphologies of (**A**) pure copper and (**B**) pure iron powders; (**C**) particle size distribution of pure copper and iron powders.

Medium-carbon steel with dimensions of 100 × 50 × 5 mm was used as the substrate for this study. Cladding copper–iron alloy on the surface of medium carbon steel can effectively improve its corrosion resistance and wear resistance. The chemical composition of medium-carbon steel is listed in Table 1. Before laser cladding, the surface of the medium-carbon steel substrate was first polished by a grinder and then cleaned in acetone and alcohol. Pure copper and iron powders with the mean particle diameter sizes of around 45.48 and 126.99 μm were employed in this study (tested by Mastersizer 3000 from Malvern Company, Malvern, UK). The morphology and distribution of particle size for these two kinds of powders are shown in Figure 1 (produced by AVIC Maite Company, Beijing, China). Cu-based monotectic alloy composite powders containing 5, 8, and 12 wt.% Fe particles were prepared by mechanical milling. Mixing powders were milled in a planetary ball miller (TJ-2L, procured from a company in Tianjin, China, TECHIN Ltd.) with 304 stainless steel balls at a ball-to-powder weight ratio of 10:1. The grinding ball consisted of two different diameter sizes of 8 and 3 mm with a proportion of 3:2; the rotation speed was 240 rpm, and the milling time was 8, 12, and 16 h, with an interval of 20 min under an Ar atmosphere. The composite powders were dried at 383K.15 for 4 h and then used as the laser cladding materials.

### 2.2. Laser Cladding Process

**Figure 2:**
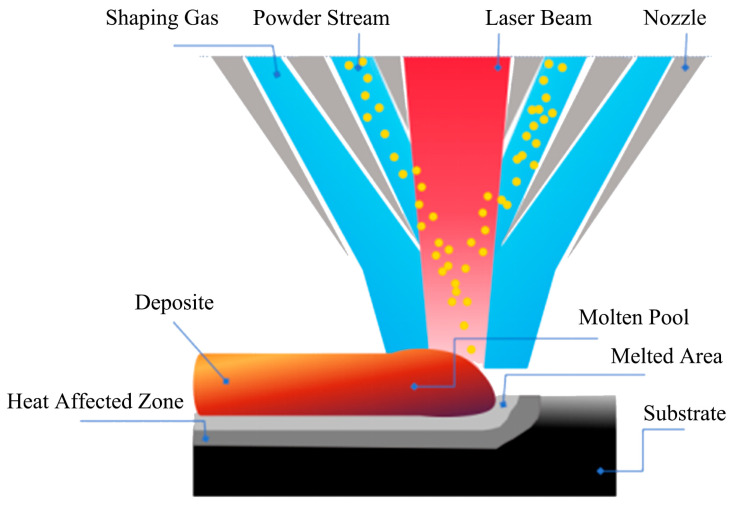
Schematic of the laser cladding process.

The laser cladding experiments were carried out using a 5 kW continuous wave CO_2_ laser (produced by Wuhan Huagong Laser Company, Wuhan, China), and the medium-carbon steel substrate was mounted on an x-y positioning table. High-purity nitrogen gas was used to protect and deliver the mixed powders into the molten pool. A series of preliminary experiments was conducted to determine the following optimal parameters: laser power of 2 kW, laser scanning speed of 200 mm/min, powder feeding rate of 0.5 g/min, laser beam spot diameter of 3 mm, and overlapping rate of 50%. The schematic configuration of the laser cladding process is depicted in Figure 2. After laser cladding, all the coatings were cut from the substrate by wire electrical discharge machining (WEDM, produced by Terui CNC Company, Taizhou, China), polished, and then etched by an FeCl_3_ solution (5 g FeCl_3_, 5 mL HCl, and 95 mL H_2_O).

### 2.3. Characterization and Test

The microstructure was examined by scanning electron microscopy (SEM) operating at 20 kV (PHILIP-XL30, produced by Philips, Netherlands) equipped with an energy dispersive analysis (EDS), and the grain size was calibrated by image software (DT2000). Phase transitions were identified using a D/MAX-2500 X-ray diffraction (XRD, Cu target, voltage 40 kV, current 40 mA) for a scanning angle range of 20° to 100° at 6°/min. Hardness was measured by a HV-1000 Vickers digital microhardness tester with a load of 1.96 N and a dwelling time of 10 s, along the cross-sectional direction away from the substrate to the top of coatings with an interval setting of 0.1 mm. The resistivity of the Cu–Fe immiscible composite coating was tested using a DC power source and a four-probe instrument with current in a range of 180 to 190 mA, and the average value was obtained via 50 times testing for each sample. The electrochemical corrosion resistance was measured by a CHI604E electrochemical analyzer (Chenhua, Shanghai, China) in a 3.5 wt% NaCl solution. A standard three-electrode cell was composed of a working electrode made from a composite specimen with an exposed area of 1 cm^2^, a platinum counter electrode, and a saturated calomel reference electrode. All the samples were immersed in the 3.5 wt% NaCl solution at room temperature for 1 h to stabilize the open circuit potential (OCP). Potentiodynamic polarization scanning was varied from −1.4 V to 1.0 V at a sweep rate of 2 mV/s. The magnetic properties were examined repeatedly using an MPMS-XL, vibrating sample magnetometer at room temperature with a maximum applied field of 20,000 Oe.

## 3. Results and Discussion

### 3.1. Morphologies of Cu–Fe Composite Powders

**Figure 3:**
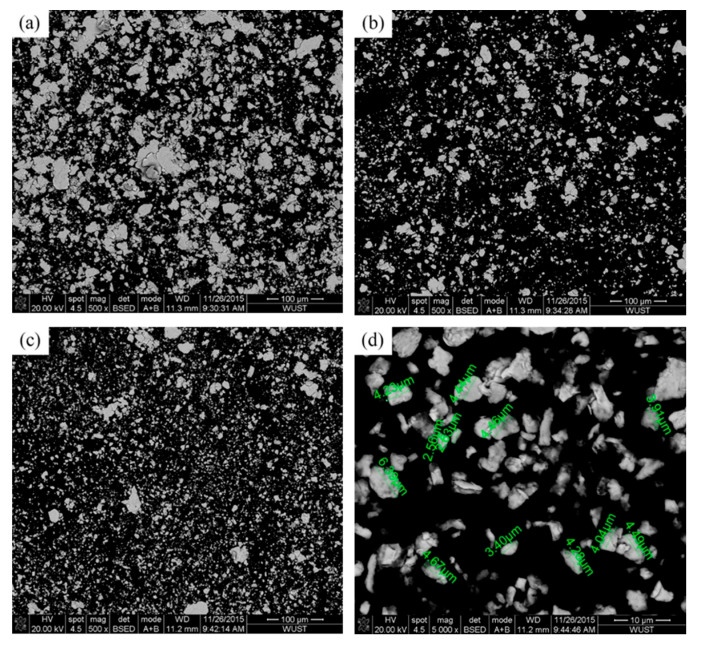
SEM micrographs of Cu95Fe5 powder: (**a**) no ball-milling, (**b**) ball-milling for 8 h [26], (**c**) ball-milling for 16 h, (**d**) ball-milling for 16 h at a higher magnification.

**Figure 4:**
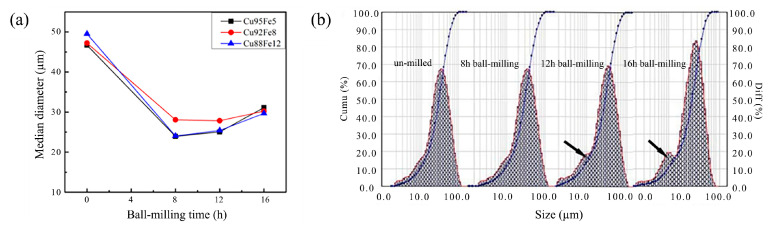
(**a**) Median diameter of Cu–Fe composite powders varies with different ball milling times; (**b**) particle size distribution of Cu92Fe8 powder varies with different ball milling times.

Figure 3 shows the microstructural features of Cu95Fe5 powder with different ball milling times. The mechanically alloyed Cu95Fe5 powders after ball milling for 8 and 16 h revealed a further refinement and polygonal morphology. The particle size and its distribution of Cu–Fe composite powders vary with different ball milling times and are presented in Figure 4. The median diameter sizes for Cu95Fe5, Cu92Fe8, and Cu88Fe12 powders without ball milling are around 50 μm and decreased significantly to 25~30 μm after ball milling for 8 h. However, this trend gradually leveled off, even slightly rebounding with continuous ball milling, as shown in Figure 4a. The D3 and D98 values of the Cu92Fe8 powder without ball milling are approximately 13.82 and 111.15 μm, respectively, and decreased significantly to approximately 5 and 80 μm, respectively, after ball milling, as shown in Figure 4b. Hence, the particle size distribution of Cu92Fe8 powder after ball milling is narrower than that without ball milling. Interestingly, the particle size distribution curve in Figure 4b shows fluctuations at the arrow mark, indicating that ball milling for 8 h or much longer can significantly decrease the particle size but also result in an uneven distribution of Cu92Fe8 powder.

The evolution of morphologies and resulting microstructures of the Cu–Fe mixed powders after ball milling can be explained by particle flattening, cold welding, and fractures [27,28]. The primary constituent of the mixed powder is Cu, which possesses low hardness but good plasticity. Initially, small particles were flattened and cold-welded to form larger aggregates. As milling progressed, fractures became the dominant mechanism, causing the particles to refine and their morphology to transition from flattened to near-spherical. Fracturing increases the particle surface area while cold welding bonds them, which facilitates the interdiffusion of alloying elements and powder refinement until the grain size reaches the sub-micro- and nanoscales [29].

### 3.2. Microstructure of Immiscible Composite Coating

**Figure 5:**
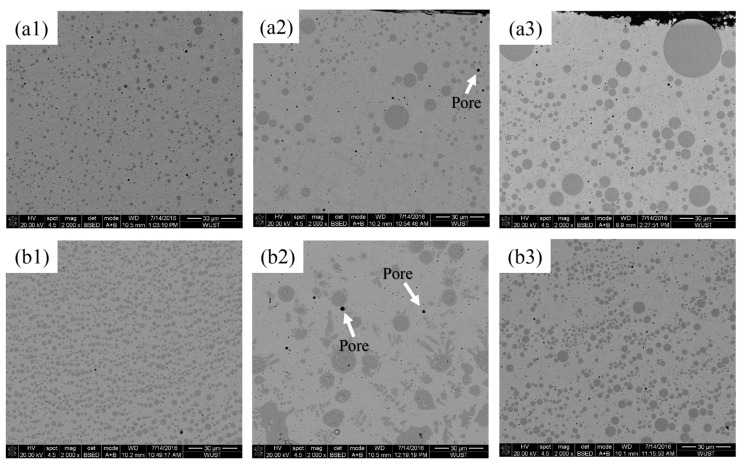
SEM images of Cu–Fe immiscible composite coatings created with layered laser cladding in cross-section. Mechanical alloying conditions are as follows: (**a1**) Cu95Fe5, (**a2**) Cu92Fe8, (**a3**) Cu88Fe12 ball milling for 8 h; (**b1**) Cu95Fe5 [26], (**b2**) Cu92Fe8, (**b3**) Cu88Fe12 ball milling for 16 h.

Microstructural features of Cu–Fe immiscible composite coatings caused by single-layer laser cladding in a cross-section with different Fe contents and ball milling times are shown in SEM images in Figure 5. The presence of spherical Fe-rich particle dispersion into a Cu-rich matrix can be observed in all coatings. With the increased Fe contents, Fe-rich phases tended to aggregate and result in large-sized segregation all over the regions, with particular severity at the top of the coatings, and also, the internal pores increased. The formation of pores is attributed to the difference in thermal expansion coefficients between the Cu and Fe elements. Due to the contraction of heterogeneous regions in rapid solidification, boundary gaps and trapping air could be introduced, leading to the formation of pores. As shown in Figure 5(a3), the presence of partially unmelted powders can be seen in the Cu88Fe12 coating with a ball milling time of only 8 h, while these phenomena almost disappeared with a much longer ball milling time of 16 h (Figure 5(b3)).

**Figure 6:**
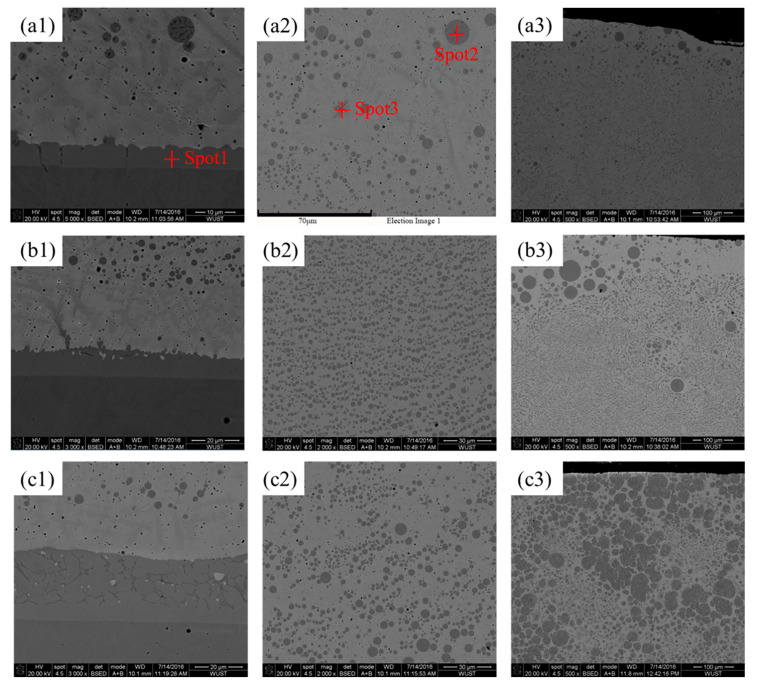
SEM images of Cu–Fe immiscible composite coatings (ball milling for 16 h) created with single-track laser cladding in cross-section: (**a1**) bottom of Cu95Fe5 coating; (**a2**) selected regions with distinctive features in (**a1**) [29]; (**a3**) top of Cu95Fe5 coating; (**b1**–**b3**) bottom, middle, and top of Cu92Fe8 coating; (**c1**–**c3**) bottom, middle, and top of Cu88Fe12 coating.

Figure 6 shows the microstructural features of Cu–Fe immiscible composite coatings (ball milling for 16 h) created through single-track laser cladding in the cross-section. As shown in Figure 6(a1,b1,c1), the presence of three distinct features can be seen at the bottom of all the Cu–Fe immiscible composite coatings. An equiaxial or columnar dendritic microstructure is formed at the combination region between the Cu–Fe immiscible composite coatings and medium-carbon steel substrate, attributed to the good diffusion between Cu and Fe. Beyond the combination region and far away from the substrate, a petal-like dendritic microstructure can be observed due to the comparable but slightly smaller extent of the diffusion. The width of the combination region in the Cu95Fe5 coating is around 6 μm (Figure 6(a1)), and the weight percentage of the Cu element is 15.88 wt.%. The EDS analysis results are shown in Table 2, indicating good diffusion between the coating and substrate since the solid solubility of Cu in Fe is very poor (less than 3%). In contrast, the widths of the combination regions in Cu92Fe8 and Cu88Fe12 increased to 11 and 33 μm, respectively (Figure 6(b2,c2)). This can be explained by Fe having a higher melting point than copper and a larger temperature gradient between the molten pool and the substrate, which encourages the Fe-rich phase to solidify first in the combination region and to form an Fe-rich region. Hence, the more Fe contents in Cu–Fe immiscible composite coatings, the more formations of Fe–rich regions, and the larger the combination region width is. The comparable higher weight percentage of Fe contents presented in spot 2 and spot 3 implies that a large number of spherical Fe-rich regions were formed throughout the coating (Figure 6(a2)).

Furthermore, large-sized spherical Fe-rich regions present an increasing tendency from the bottom to the top of the Cu–Fe immiscible composite coatings, as shown in Figure 6(b2,b3,c2,c3). The possible reason could be the effects of Marangoni motion induced by the temperature gradient and surface tension gradient between spherical liquid droplets and liquid matrix, as well as Stokes motion caused by the gravity field. The liquid droplets can grow by consuming the smaller liquid droplets due to Ostwald ripening and coalescence through the collisions of liquid droplets [30,31,32]. The velocities of Marangoni motion, $V_{M}$, can be determined by the following equations [33]:

(1) $$V_{M}\;=\;\frac{k\times \Delta \sigma }{(2k+k^{\prime })(2\eta +3\eta^{\prime })}\cdot d$$

where *k* and *k′* are the thermal conductivities of the matrix and droplet, respectively; *η* and *η′* are the viscosities of the matrix and droplet, respectively; *d* is the droplet diameter; and Δσ is the surface tension gradient between the interface of the matrix and the droplet. From Equation (1), it can be seen that the droplet Marangoni motion is affected by the surface tension gradient and droplet diameter. With the increased droplet diameter, the droplet motion speed can be improved, prompting the precipitation of a large-sized Fe-rich phase to float to the top of the coating. The Stokes motion velocity, $V_{s}$, can be calculated as

(2) $$\mathrm{Vs}=\frac{d^{2}\times (\rho^{\prime }-\rho )(\eta +\eta^{\prime })\times g}{6\eta \times (2\eta +3\eta^{\prime })}$$

where *ρ* and *ρ′* are the densities of the substrate and droplet, respectively, and *g* is the gravitational acceleration. From Equation (2), it can be seen that the droplet Stokes motion is related to the density difference and droplet diameter, so the large-sized precipitated phase tends to move toward the bottom of the coating through the effects of Stokes motion. Thus, the movement velocity of precipitated droplets depends on the ratio of $V_{m}$ to $V_{s}$, that is,

(3) $$\frac{V_{M}}{V_{S}}=\frac{6k}{2k+k^{\prime }}\cdot \frac{\eta }{\eta +\eta^{\prime }}\cdot \frac{\Delta \sigma }{(\rho^{\prime }-\rho )g}\cdot \frac{1}{d}$$

From Equation (3), it can be seen that when the ratio of $\frac{V_{M}}{V_{S}}$ is greater than 1, Marangoni motion plays a dominant role in the determination of the droplets’ movement toward the top of the coating, while when the ratio is less than 1, Stokes motion plays a dominant role in the determination of the droplets’ movement that toward the bottom of the coating. $\frac{V_{M}}{V_{S}}$ was calculated to be 1 at a droplet radius of 38.1 μm based on data from the literature [34]. Consequently, droplets with a diameter size less than 38.1 μm in Figure 6 are not sufficiently large to move either toward the top or bottom of the coating and would be retained in the middle of the coating.

**Figure 7:**
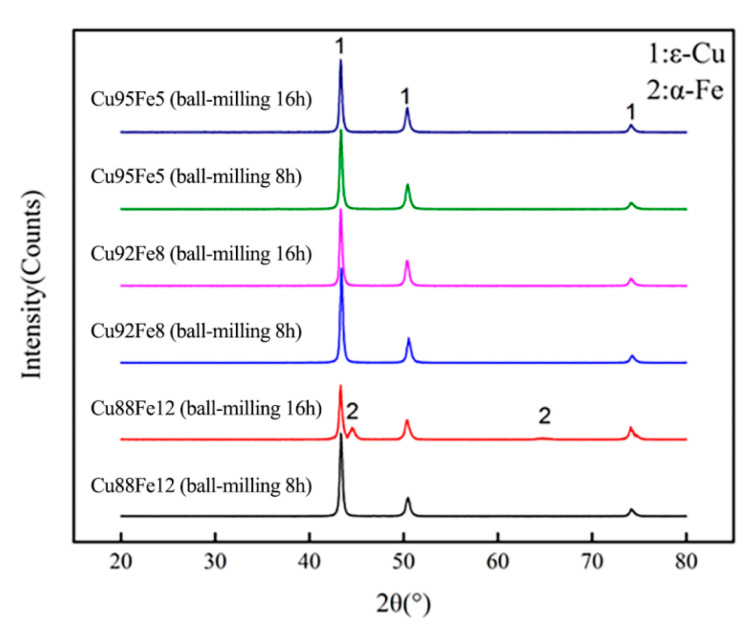
XRD patterns of Cu95Fe5, Cu92Fe8, and Cu88Fe2 immiscible composite coatings with ball milling for 8 and 16 h.

Figure 7 shows the XRD patterns of the Cu95Fe5, Cu92Fe8, and Cu88Fe2 immiscible composite coatings with ball milling for 8 and 16 h. It can be seen that the face-centered cubic *ε*-Cu phase was detected in all coatings, but not for the body-centered cubic *α*-Fe phase; this is attributed to the dispersion of the Fe element in the matrix and the formation of fine particles.

### 3.3. Hardness

**Figure 8:**
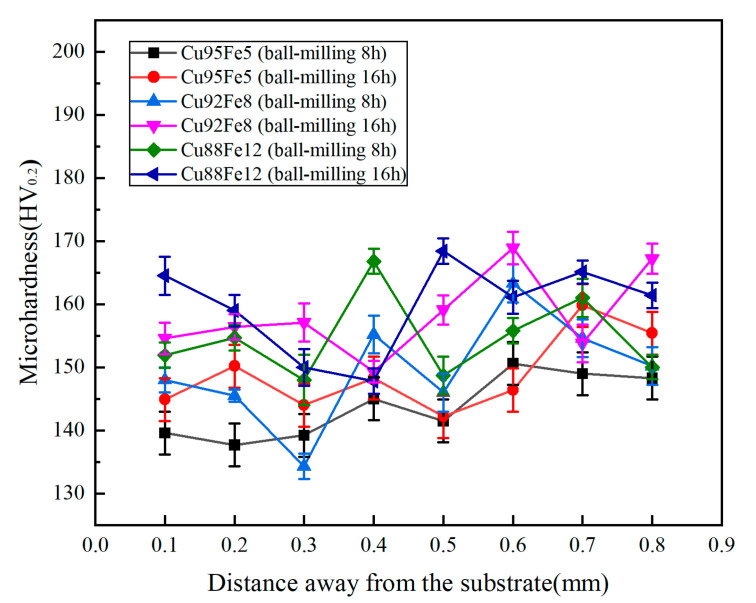
Hardness results for coatings with varying compositions and ball milling times.

Figure 8 shows the microhardness of the Cu–Fe immiscible composite coatings with varying compositions and ball milling times. Each point is tested three times to obtain an average value. It can be seen that the variation in microhardness across the combination area is smaller, indicating homogeneous microstructures and element distribution across the combination region. The microhardness values of the Cu95Fe5, Cu92Fe8, and Cu88Fe12 coatings with ball milling of 8 h are 143.6, 149.5, and 154.2 HV_0.2_, respectively, and the microhardness values of the Cu95Fe5, Cu92Fe8, and Cu88Fe12 coatings with ball milling of 16 h are 149.1, 157.8, and 158.0 HV_0.2_, respectively, much higher than that of pure copper (106 HV_0.2_). This is attributed to the number of spherical Fe-rich particles dispersed within the Cu-rich matrix. The dispersion strengthening follows Orowan’s theory and is governed by the equation below:

(4) $$\Delta \tau \;=\;0.81\frac{\mathrm{Gb}}{2\pi }\times \frac{1}{\sqrt{\left(1-\mu \right)}}\times \frac{1}{\lambda -d}\ln\left(d/r_{0}\right)$$

where G is the shear elastic modulus of the matrix phase, b is the Berber vector, μ is Poisson’s ratio, d is the particle diameter, λ is the particle spacing, and $r_{0}$ is the radius of the dislocation line. From Equation (4), it can be seen that the narrowing of the particle spacing facilitates the enhancement of the strengthening effect, and the increased Fe content promotes the amount of Fe phase precipitation, which can just narrow the precipitation phase spacing, leading to an increase in microhardness. By increasing the ball milling time from 8 to 16 h, the extent of dispersion of Fe-rich particles in the Cu-rich matrix becomes larger, resulting in a slight increase in hardness for the coating with ball milling of 16 h compared with that of the coating with ball milling of 8 h.

### 3.4. Electrical Resistivity

**Figure 9:**
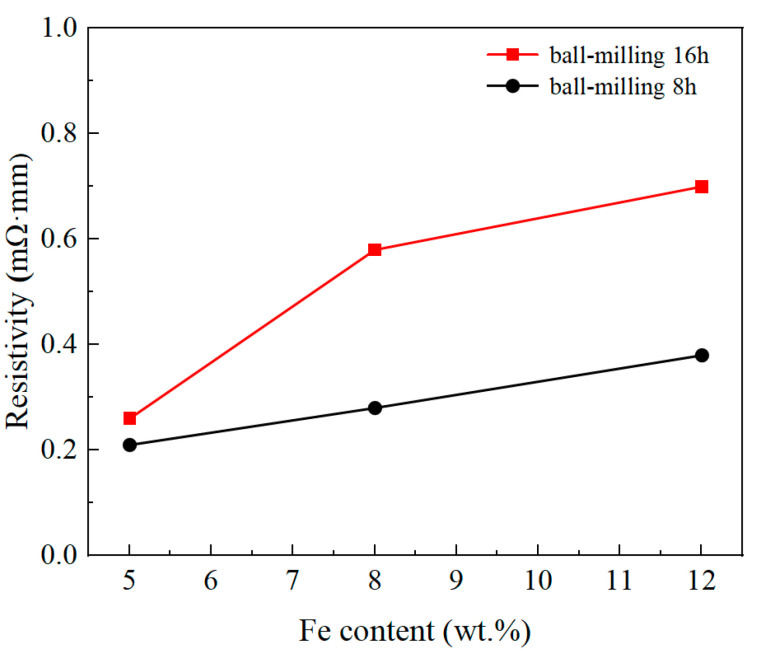
Resistivity of coatings with varying compositions and ball milling times.

Figure 9 shows the resistivity changes in the Cu–Fe immiscible composite coatings with varying compositions and ball milling times. It can be observed that the resistivity of the Cu–Fe immiscible composite coatings increased as more Fe content was added. The electrical conductivity of pure copper at room temperature is 1.7 × 10^−2^ mΩ·mm, which decreased dramatically with elemental doping. According to Hong et al. [34], the overall resistivity of pure copper is related to the individual resistivity, volume fraction, size, and distribution of its constituent phases. The individual resistivity of the constituent phases can be expressed as

(5) $$\rho_{x}=\rho_{\mathrm{pho}}+\rho_{\mathrm{imp}}+{\;\rho }_{\mathrm{dis}}+\rho_{\mathrm{int}}$$

where $\rho_{\mathrm{pho}}$, $\rho_{\mathrm{imp}}$, $\rho_{\mathrm{dis}}$, and $\rho_{\mathrm{int}}$ represent the resistivity caused by phonon scattering, impurity scattering, dislocation scattering, and interface scattering, respectively. Phonon scattering is an inherent property of the material and is temperature-dependent, while impurity scattering is caused by impurity elements dissolved in the Cu-rich matrix. Thus, the dispersion of Fe-rich particles in the Cu-rich matrix results in an increase in resistivity. The resistivity of the coating with ball milling of 16 h is larger than that of the coating with ball milling of 8 h. This is attributed to the increase in the median particle size of the powder shown in Figure 3. Larger particle sizes are detrimental to the dispersion of heterogenous elements after cladding, and the segregation of heterogenous phases disrupts the continuity of the copper matrix, causing an increment in the resistivity of the coatings.

### 3.5. Electrochemical Corrosion

**Figure 10:**
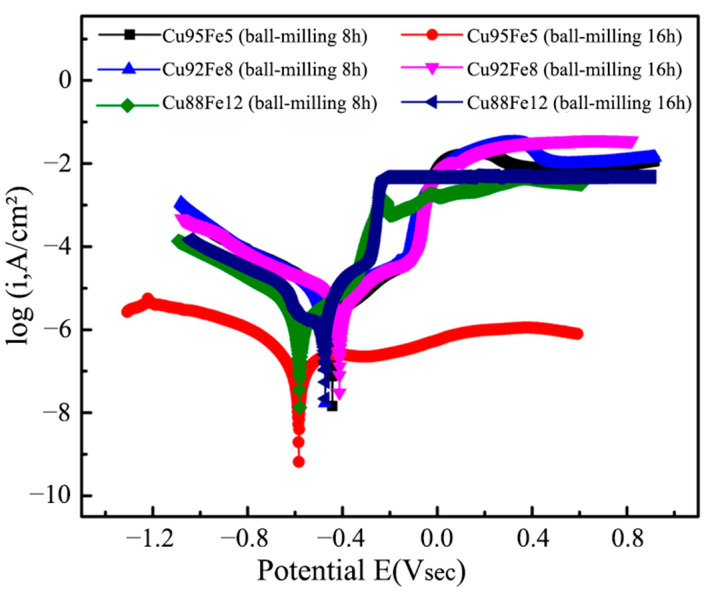
Potential dynamic curves of the Cu–Fe immiscible coatings in the 3.5 wt% NaCl solution.

Figure 10 shows the anodic polarization curves of Cu–Fe immiscible composite coatings with varying compositions and ball milling times in 3.5% NaCl solution at room temperature. The corrosion potential (E_corr_) and corrosion current density (I_corr_) according to the intersection of the cathode and anode polarization curves were calculated by the Tafel extrapolation method from the respective curves summarized in Table 3. Generally, the corrosion thermodynamics and corrosion rate of a material can be reflected by the corrosion potential, E_corr_, and current density, I_corr,_ respectively. With greater corrosion potential, E_corr_, the corrosion trend is lower, while the corrosion rate is sped up with a larger current density, I_corr_. All the Cu–Fe immiscible coatings with different compositions have a much higher E_corr_ (larger than −0.600 V) and a smaller I_corr_ (less than 6.000 × 10^−6^ A/cm^2^) than the medium-carbon steel substrate (−0.955 V and 1.973 × 10^−5^ A/cm^2^) [26]. This indicates that the Cu–Fe immiscible coatings significantly improved the corrosion resistance of the medium-carbon steel substrate. Moreover, the E_corr_ decreased, but I_corr_ increased with the increased fractions of Fe contents in the Cu–Fe immiscible coatings, while the trends with the increased ball milling times are the reverse. This indicates that adding more Fe to Cu–Fe immiscible coatings results in a gradual weakening of the corrosion resistance, while adequate and proper longer ball milling time is beneficial to improving the corrosion resistance. The corrosion resistance of the Cu–Fe immiscible composite coatings is higher than that of the medium-carbon steel substrates, attributed to the formation of microcells caused by potential differences between spherical Fe-rich particles and the copper substrate. Specifically, the high-potential Cu-rich regions and low-potential Fe-rich regions act as large cathodes and small anodes, respectively. Thus, the Fe-rich particles are preferentially corroded, thereby protecting the copper matrix from corrosion. Moreover, bubbles appeared during the electrochemical testing process, indicating that hydrogen gas was released on the cathode side during the corrosion process. The relevant reaction equations can be described as follows:

(6) $${2H}_{2}O+{2e}^{-}\;\to \;2{\mathrm{OH}}^{-}+H_{2}\;↑$$

(7) $$\mathrm{Fe}+{2\mathrm{Cl}}^{-}\;\to \;\mathrm{Fe}{\mathrm{Cl}}_{2}+2e^{-}$$

(8) $$4\mathrm{Fe}{\mathrm{Cl}}_{2}+{8\mathrm{OH}}^{-}+O_{2}\;\to \;2{\mathrm{Fe}}_{2}O_{3}\cdot {2H}_{2}O+8\;{\mathrm{Cl}}^{-}+{2H}_{2}$$

Adding more Fe contents induces more Fe-rich particles distributed at the top of the Cu–Fe immiscible composite coatings (Figure 6), resulting in more intense microcell reactions during electrochemical corrosion and thereby degrading the corrosion resistance. Ball milling for a longer time can significantly facilitate the refinement of Fe-rich particles inside these coatings, impeding their Marangoni and Stokes motion, which in turn results in a more concentrated distribution of Fe-rich particles at the middle rather than at the top of the coatings. Therefore, the microcell reactions at the top of the coatings are suppressed during the electrochemical corrosion and thereby enhance the corrosion resistance. Interestingly, the Cu95Fe5 coating with ball milling of 16 h has a lower current density, and its corrosion potential is also much smaller than that of the Cu92Fe8 and Cu88Fe12 coatings with ball milling of 16 h. A lower corrosion current density reflects a higher corrosion resistance for the Cu95Fe5 coating, and its lower corrosion potential could be attributed to the segregation and concentrated distribution of Fe-rich particles at the top of the coating.

### 3.6. Magnetic Hysteresis Loop Analysis

**Figure 11:**
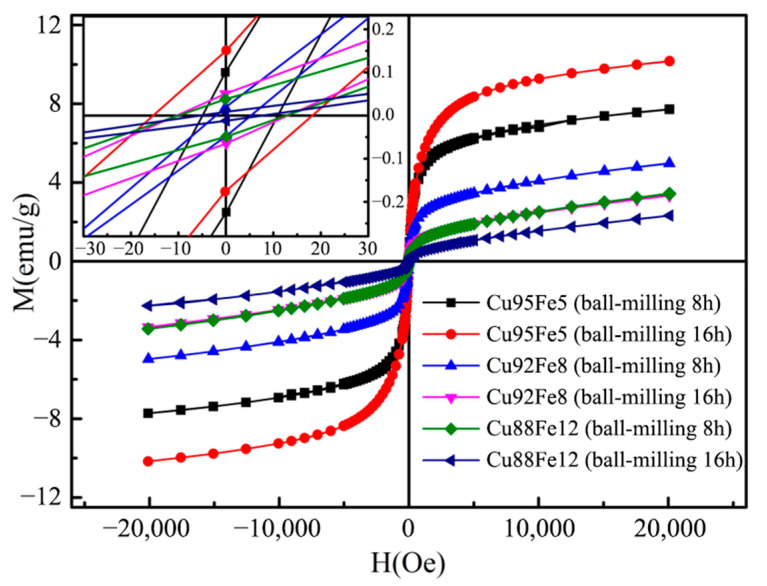
Magnetic hysteresis loop of the Cu–Fe immiscible coatings.

Figure 11 shows the magnetic hysteresis loop of Cu–Fe immiscible composite coatings with varying compositions and ball milling times, and the fitted values of the magnetic hysteresis loops are given in Table 4. The values of the saturated magnetization and coercivity of the Cu88Fe12 coating with ball milling of 8 h are 3.348 emu/g and 1.756 Oe, respectively. Liu et al. [35] produced immiscible Cu-Fe alloys with variation in Fe content through vacuum arc melting, which possessed a saturated magnetization of 76.8216 emu/g and a coercive force of 24.613 Oe. Therefore, the Cu–Fe immiscible composite coating presents excellent soft ferromagnetic characteristics of low coercivity and high permeability, which can be expected to have good applications in power electronics and electrical machines (motors, generators, etc.). Meanwhile, increased iron content further promotes a reduction in the coercivity of copper–iron alloy coatings. The coercive force is related to the size and distribution of Fe-rich particles in the immiscible composite coatings. Refined and uniformly distributed Fe-rich particles may decrease coercive force due to the size effect. Moreover, according to our previous research [36], the introduction of reinforcing ceramic phases such as TiB_2_ and Fe_2_P hinders the Marangoni and Stokes motion of iron-rich droplets, thereby reducing the size of Fe-rich particles that possess very unstable magnetic moments, which in turn results in a decrease in the overall saturation magnetization strength.

## 4. Conclusions

Cu–Fe immiscible composite coatings were successfully prepared by mechanical alloying and laser cladding. The microstructure, microhardness, electrical resistivity, electrochemical corrosion performance, and magnetic properties of Cu–Fe immiscible composite coatings with varying compositions and ball milling times were systematically investigated. The addition of more Fe content can facilitate the formation of much larger-sized segregations of spherical Fe-rich particles in Cu–Fe immiscible composite coatings, resulting in an increment in microhardness and electrical resistivity, as well as a degradation in corrosion resistance. Ball milling had a significant influence in improving the distribution of Fe-rich particles in the Cu-rich matrix and hence significantly affects the comprehensive performance of the Cu–Fe immiscible composite coatings. With an increased ball milling time, the electrical resistivity increases while the corrosion resistance is improved. In addition, soft ferromagnetic characteristics of low coercivity and high permeability for the Cu–Fe immiscible composite coatings were achieved. Thus, combined fabrication via mechanical alloying and laser cladding caused the Cu–Fe immiscible composite coatings to perform better.

## Figures and Tables

**Table 1:** Chemical composition of medium-carbon steel substrate.

Element	C	Mn	Si	S	P	Fe
Composition (wt.%)	0.22	0.14	0.35	0.05	0.045	Bal.

**Table 2:** EDS analysis results of spots 1~3 in the Figure 6.

Location	Composition (wt.%)					
	P	Cr	Fe	Ni	Cu	Zn
Spot 1	0	6.15	60.02	17.95	15.88	0
Spot 2	2.59	7.55	53.63	20.98	15.25	0
Spot 3	0	7.10	58.57	19.64	14.69	0

**Table 3:** Corrosion parameters of the Cu–Fe immiscible coatings in Figure 10.

Sample	Ball-Milling Time (h)	E_corr_ (V)	I_corr_ (A/cm^2^)
Cu95Fe5	8	−0.444	1.906 × 10^−6^
Cu92Fe8	8	−0.473	4.818 × 10^−6^
Cu88Fe12	8	−0.581	5.895 × 10^−6^
Cu95Fe5	16	−0.585	0.936 × 10^−6^
Cu92Fe8	16	−0.413	3.908 × 10^−6^
Cu88Fe12	16	−0.471	5.849 × 10^−6^

**Table 4:** Hysteresis loop fitting values.

Sample	Ball Milling Time (h)	Saturated Magnetization (emu/g)	Remnant Magnetization (emu/g)	Coercive Force (Oe)
Cu95Fe5	8	7.723	0.175	9.858
Cu92Fe8	8	4.980	0.046	2.313
Cu88Fe12	8	3.348	0.043	1.756
Cu95Fe5	16	10.172	0.163	17.249
Cu92Fe8	16	3.342	0.059	11.053
Cu88Fe12	16	2.319	0.010	4.983

## Data Availability

The original contributions presented in this study are included in the article. Further inquiries can be directed to the corresponding author.
